# Early Osseointegration and Lower Loosening Rates With Trabecular Metal Revision Cups in Acetabular Bone Loss: A Comparative Cohort Study

**DOI:** 10.7759/cureus.97524

**Published:** 2025-11-22

**Authors:** Monica Georgiana Roman, Alexandru Lisias Dimitriu, Elisa Georgiana Popescu, Eduard Catalin Georgescu, Razvan Ene, Dragos Ene

**Affiliations:** 1 Orthopaedics, Carol Davila University of Medicine and Pharmacy, Bucharest, ROU; 2 Orthopaedics and Trauma, Clinical Emergency Hospital, Bucharest, ROU; 3 Surgery, Carol Davila University of Medicine and Pharmacy, Bucharest, ROU; 4 Surgery, Clinical Emergency Hospital, Bucharest, ROU

**Keywords:** acetabular bone loss, cup fixation, loosening, osseointegration, paprosky iic–iii, revision tha, trabecular metal

## Abstract

Background: Revision total hip arthroplasty (THA) in the presence of significant acetabular bone loss remains challenging, particularly in Paprosky IIC-III defects, where biomechanical stability and biological fixation are often compromised. Trabecular metal (TM) revision cups were developed to enhance osseointegration through high porosity and an elastic modulus closer to native bone, yet comparative real-world evidence against conventional revision cups remains limited in early follow-up.

Methods: We conducted a retrospective comparative cohort analysis of patients undergoing acetabular revision THA for Paprosky IIC-III defects between 2022 and 2024. Ten patients were included, with six receiving TM cups (TM group) and four receiving conventional revision cups (control group). Radiographic osseointegration was assessed through secondary signs of ingrowth (absence of radiolucent lines, bone apposition, and absence of migration), while clinical fixation and early stability were evaluated using postoperative Harris Hip Score (HHS) and complication rates. Minimum follow-up was 12 months.

Results: Early radiographic osseointegration was observed in 100% of the TM group versus 50% of the conventional group at 12 months. No cup migration or radiographic loosening occurred in the TM group, while two cases in the control group demonstrated early signs of mechanical instability. Mean postoperative HHS improved more robustly in the TM group (from 41.8 to 78.6) compared to controls (from 40.9 to 68.3). There were no infections or dislocations in either cohort. One patient who received a conventional cup required prolonged protected weight bearing due to suspected partial loss of early fixation.

Conclusion: In revision THA for Paprosky IIC-III acetabular defects, TM cups demonstrated markedly higher rates of early osseointegration and lower radiographic loosening at 12 months compared with conventional revision cups. These findings support the preferential use of TM technology in cases of advanced acetabular bone loss, where biological fixation is critical for long-term stability.

## Introduction

Revision total hip arthroplasty (THA) in the setting of significant acetabular bone loss remains one of the most demanding procedures in reconstructive hip surgery. The loss of native bone stock compromises both initial fixation and long-term biological stability, particularly in Paprosky IIC-III defects, where conventional revision cups often struggle to achieve a durable press-fit or reliable ingrowth. As the burden of revision arthroplasty continues to grow worldwide, the success of acetabular reconstruction increasingly depends on materials capable of restoring both mechanical and biological integrity.

Traditional revision acetabular components rely primarily on structural fit and supplemental screw fixation to achieve short-term stability. However, when the host bone is insufficient or severely compromised, even well-positioned cementless revision cups may exhibit micromotion beyond the threshold tolerated for osseointegration. This instability contributes to persistent radiolucent lines, incomplete bony ingrowth, and ultimately early loosening, especially in cases of medial wall erosion or major segmental deficiency. The challenge is therefore not only reconstructive but also biological, because fixation must transition from purely mechanical support to biologically anchored integration.

Trabecular metal (TM) technology was developed precisely to address these limitations. Its high volumetric porosity and low elastic modulus create an interface environment far more permissive for bone ingrowth compared to conventional porous coatings. The structural “scaffold-like” architecture promotes deep bony infiltration, while its friction coefficient enhances initial stability even in compromised bone. The theoretical advantages are well recognized, yet their clinical translation is most evident in cases with large acetabular defects, where conventional revision cups frequently underperform.

Despite growing use of TM constructs, comparative evidence in severe acetabular bone loss remains limited, and most available reports cluster TM implants together with other porous metals or evaluate them in mixed-problem populations. Few studies isolate revision cases with major Paprosky IIC-III defects and directly compare early osseointegration and loosening rates with conventional revision cups. This gap is clinically relevant because it is precisely in high-grade bone loss where biological integration matters most and determines long-term survivorship.

The present retrospective comparative cohort study focuses exclusively on revision THA patients with Paprosky IIC-III bone defects and evaluates early fixation and osseointegration outcomes between TM cups and conventional revision acetabular cups. Our rationale is that if the material truly confers a biologic advantage, this difference should be detectable early, even within the first 12 postoperative months, through radiographic signs of ingrowth and the absence of micromotion.

We hypothesize that TM cups demonstrate faster and more reliable osseointegration, with lower early loosening rates compared with conventional revision cups in the context of severe acetabular bone loss. Confirming this advantage would reinforce the preferential indication of TM constructs in reconstructive scenarios where the biologic environment is compromised and durable secondary fixation is critical for long-term success.

## Materials and methods

Study design

This was a retrospective comparative cohort study evaluating early fixation and radiographic osseointegration in revision THA in the setting of acetabular bone loss. Two groups were analyzed: patients receiving TM revision cups (TM group) and those receiving conventional cementless revision cups (control group). All procedures were performed at a single orthopedic center between 2022 and 2024.

Patient selection

Patients were eligible for inclusion if they underwent acetabular revision THA for Paprosky IIC-III acetabular defects confirmed preoperatively through standard radiographs and CT-based evaluation, where available. Exclusion criteria included septic revisions, periprosthetic fractures requiring structural allograft reconstruction, and cases with incomplete radiographic follow-up at 12 months. Ten patients met the inclusion criteria, with six assigned to the TM group and four to the control group. Implant selection was determined intraoperatively according to bone stock quality and implant availability, minimizing selection bias between groups.

Ethical approval

Institutional review board approval was obtained before data collection. The study was conducted in accordance with the Declaration of Helsinki, and all data were anonymized prior to analysis.

Surgical technique

All revisions were performed through a posterior approach. All procedures were performed by the same senior orthopedic surgeon using a consistent surgical approach and fixation strategy, minimizing inter-operator variability. Cup sizing and implantation technique followed standard reconstructive principles to achieve maximal host bone contact. In the TM group, primary press-fit stability was pursued with supplemental screw fixation according to intraoperative assessment of native bone purchase. In the conventional group, cementless revision cups with porous coating were implanted similarly, using adjunct screw fixation as required. Only reconstructions without cages or augments were included to avoid confounding variables in the evaluation of biological fixation.

Postoperative protocol

Patients in both cohorts received the same postoperative rehabilitation strategy, consisting of early mobilization with partial weight bearing for the first four to six weeks, advancing to full weight bearing as tolerated thereafter. Clinical and radiographic follow-up was performed at 6 weeks, 3 months, 6 months, and 12 months.

Radiographic assessment of osseointegration

Radiographic fixation and early osseointegration were evaluated through predefined objective criteria: absence of radiolucent lines at the bone-implant interface, presence of bone apposition or trabecular bridging adjacent to the implant, and absence of cup migration or positional change on serial imaging.

The presence of all three criteria was considered evidence of successful osseointegration. Cup loosening was defined as progressive radiolucency, implant migration, or mechanical instability observed on serial radiographs.

Clinical outcomes

Patient function was assessed using the Harris Hip Score (HHS), recorded preoperatively and at final follow-up (12 months). The HHS is a freely available and non-proprietary clinical outcome tool originally developed by Harris in 1969 and does not require licensing for academic use [1]. Complications, including dislocation, infection, and mechanical failure, were also documented.

Data handling and statistical approach

Given the small cohort size, outcomes were reported using descriptive statistics rather than inferential tests. The analysis focused on comparative trends in early biological fixation and loosening rates between the two implant types.

## Results

Patient characteristics

A total of 10 patients were included, with six in the TM group and four in the conventional revision cup group. The mean age at surgery was comparable between groups (TM: 69.1 years; control: 67.4 years). All patients presented with Paprosky IIC-III acetabular defects. No patients were lost to follow-up at 12 months.

Radiographic outcomes

Successful early osseointegration, defined by the presence of bone apposition, absence of radiolucent lines, and no implant migration, was observed in all six patients (100%) in the TM group at 12 months. In the control group, only two out of four patients (50%) demonstrated complete radiographic signs of early osseointegration. The remaining two showed progressive radiolucent lines suggestive of early mechanical compromise.

No cup migration was detected in the TM cohort, whereas two cups in the control group demonstrated early positional change consistent with partial loss of primary fixation.

Clinical outcomes

Mean HHS improved from 41.8 to 78.6 in the TM group and from 40.9 to 68.3 in the control group at final follow-up. TM patients reported earlier pain reduction and functional recovery, with more reliable progression toward full weight bearing.

Complications

There were no postoperative infections or dislocations in either group. One patient in the control cohort required prolonged protected weight bearing due to radiographic suspicion of insufficient biological fixation.

Comparative summary

TM implants demonstrated a clear advantage in both radiographic and functional outcomes at 12 months, with consistently higher rates of early biological fixation and no early loosening events.

These findings are summarized in Table 1, which illustrates the comparative osseointegration and fixation profile between TM and conventional revision cups.

**Table 1:** Early osseointegration and fixation outcomes in trabecular metal versus conventional revision cups (12-month follow-up) HHS: Harris Hip Score [1]. Paprosky classification from ref [2].

Parameter	Trabecular Metal (n = 6)	Conventional Cup (n = 4)
Paprosky type	IIC–III	IIC–III
Early osseointegration (12 months)	100%	50%
Radiolucent lines	0	2
Cup migration	0	2
HHS (pre → 12 months)	41.8 → 78.6	40.9 → 68.3
Early loosening	0	2

## Discussion

The findings of this study demonstrate a clear advantage of TM acetabular cups over conventional revision cups in the setting of Paprosky IIC-III defects, particularly with regard to early biological fixation. All patients in the TM group exhibited radiographic signs of osseointegration at 12 months, whereas half of the conventional implants showed progressive radiolucency suggestive of early mechanical compromise. Although the cohort size is limited, the consistency of fixation outcomes in the TM group indicates that the material properties of these implants translate into clinically meaningful early stability, even in severe acetabular bone loss.

A likely explanation for these findings is the improved primary stability afforded by TM constructs. In the setting of compromised acetabular bone stock, press-fit fixation alone can be unreliable when using standard revision cups, which often depend on intact rim or column support to limit micromotion. TM implants provide a higher friction interface and a more conforming surface architecture, improving initial implant-bone contact and reducing micromovement during the early postoperative phase. This mechanical advantage is critical because excessive micromotion at the bone-implant interface has been shown to inhibit bone ingrowth and promote fibrous encapsulation rather than osseous incorporation.

Beyond primary stability, the biological environment created by TM interfaces also favors secondary fixation. The high volumetric porosity increases the available surface area for bone apposition and facilitates deeper structural integration rather than superficial ingrowth. This scaffold-like configuration mimics cancellous bone and permits more reliable penetration of bridging trabeculae, which supports the development of a stable, biologically anchored interface. In contrast, conventional porous-coated cups rely more heavily on host bone quality and do not replicate the same three-dimensional microarchitecture, making them more vulnerable to incomplete integration in the presence of segmental or cavitary loss.

The correlation observed in this study between radiographic osseointegration and functional recovery further supports the biological superiority of TM in major acetabular defects. Patients receiving TM cups not only showed earlier radiographic signs of fixation but also achieved higher functional scores at 12 months. This suggests that secure biological accretion facilitates earlier and more confident mobilization, which in turn enhances rehabilitation and overall clinical outcome.

The role of implant surface properties in achieving early fixation has also been highlighted in previous work by the authors, where biotribological characteristics of different bearing surfaces were shown to influence the stability of the host-implant interface [3].

Revision THA in the setting of severe acetabular bone loss remains biomechanically and biologically challenging, and the results of this study highlight a clear early fixation advantage of TM acetabular cups over conventional revision cups in Paprosky IIC-III defects. All implants in the TM group demonstrated complete radiographic osseointegration at 12 months, whereas only half of the conventional revision cups achieved similar integration. This divergence in early fixation profile suggests that, in the presence of compromised bone stock, the material characteristics of TM play a direct role in enabling stable ingrowth and preventing micro-instability during the biologically decisive early postoperative phase. These findings align with the growing evidence that early micromotion thresholds strongly influence long-term implant survival in revision hip arthroplasty [4, 5].

A key factor contributing to the superior performance of TM constructs appears to be enhanced primary stability. In revision settings, especially when acetabular rim support is deficient, mechanical fixation through a classical press-fit becomes unreliable and vulnerable to micromotion. Conventional revision cups rely substantially on host cortical continuity to achieve sufficient grip, which is often lacking in IIC-III defects. By contrast, the macrostructure and conforming surface geometry of TM facilitate broader contact distribution along the remaining viable acetabular segments, translating into improved initial stability under load [6]. Reduction of micromotion during the early bone healing window is critical, as exceeding tolerated thresholds promotes fibrous tissue interposition rather than osseous anchorage [7].

Equally important is the biological environment created by the three-dimensional pore configuration of TM implants. Unlike traditional porous-coated cups, which permit primarily surface-level ingrowth, the architecture of ultra-porous metal scaffolds supports a deeper and more continuous bone ingrowth pathway, enabling trabecular penetration rather than mere appositional adherence [8]. This internal colonization of the scaffold enhances secondary stability, which is particularly advantageous when host bone stock is compromised. In other words, these implants not only tolerate imperfect bone bed quality but also biologically compensate for it by providing a more permissive matrix for osseointegration [9]. The reliability of fixation observed in this study, therefore, reflects a dual mechanism: improved initial mechanical capture combined with accelerated biological consolidation.

Additionally, the correlation between radiographic osseointegration and clinical functional improvement reinforces the notion that early stability is not merely a radiologic marker but a determinant of patient recovery dynamics. In the present cohort, patients with TM implants achieved higher functional scores at 12 months, suggesting that a stable biological interface enables earlier loading confidence and rehabilitation progression. This aligns with the concept that implant biology and postoperative functional trajectory are interdependent rather than sequential phenomena [10].

These results are consistent with previous reports demonstrating that high-porosity metal constructs achieve more predictable fixation in revision acetabular reconstruction, particularly when conventional press-fit designs are limited by deficient host support [4,6]. Several clinical studies have shown improved radiographic survival of TM implants in high-grade bone loss compared to standard porous revision cups, especially during the first 12-24 months when biological integration is most vulnerable to micromotion [7]. The present findings strengthen this trend by demonstrating that the divergence in outcomes emerges early and is already measurable at one-year follow-up, which supports the concept that material-driven differences in integration are clinically relevant from the initial postoperative phase onward.

From a mechanistic standpoint, previously published work in biomechanical models has shown that high-porosity metal interfaces reduce interface shear and facilitate deeper marrow-derived vascular penetration, two prerequisites for stable osseous anchorage in compromised bone environments [9,10]. These properties appear particularly advantageous in Paprosky IIC-III defects, where native cancellous structure has been partially or completely lost and where conventional revision components depend disproportionately on rim fixation. In such cases, the ability of TM to create a biologically favorable scaffold substitutes for the absent trabecular substrate that conventional cups rely upon.

The clinical implications of these findings extend beyond radiographic performance. Stable early osseointegration reduces the need for prolonged protected weight bearing and facilitates earlier functional recovery. This is consistent with prior institutional experience emphasizing the role of biological environment and systemic host factors in post-fracture healing dynamics [11], as previously demonstrated by the authors in reconstructive populations [12]. The concordance between biological permissiveness and functional rehabilitation seen in our cohort reinforces the relevance of implant selection not only as a mechanical choice but as a biologically strategic one.

These results are clinically relevant in the context of patients with significant medical comorbidities, where systemic factors further compromise bone quality and rehabilitation potential, as previously reported in a multicenter analysis of periprosthetic fracture patients [13]. 

This observation is consistent with population-level data showing that patient health status is a major determinant of revision outcomes and early postoperative complication risk in THA patients [14]. 

These reconstructive considerations align with broader revision strategies described in contemporary acetabular reconstruction literature, which emphasize implant selection based on host bone biology and defect morphology rather than cup size alone [15]. 

Large-scale registry data also support this observation, with propensity score-matched analysis from the National Joint Registry for England and Wales showing a significantly lower risk of revision when TM acetabular components are used compared with conventional cups [16].

This study has limitations. The cohort size is small and reflects the real-world frequency of severe acetabular defects within a single institution. These are technically demanding revision procedures that are not frequently encountered, which explains the restricted sample size. The follow-up interval is limited to 12 months, and although this captures the phase of biological fixation, it does not address long-term survivorship. Additionally, cases reconstructed with augments or cages were excluded to isolate the effect of the acetabular cup alone. Nevertheless, this homogeneity strengthens the internal validity of the observed fixation patterns and underscores that the material advantage of TM is detectable even under strictly matched conditions.

In summary, the findings of this comparative analysis indicate that TM revision cups achieve more reliable early osseointegration than conventional porous-coated revision cups in the setting of Paprosky IIC-III acetabular defects. This observation is consistent with prior biomechanical and clinical studies demonstrating that high-porosity tantalum constructs provide enhanced primary stability and facilitate early bone ingrowth, as described by Bobyn et al. [17]. By providing both enhanced primary stability and a biologically permissive scaffold for secondary fixation, TM implants appear to mitigate the consequences of compromised host bone structure and support more consistent early integration. These results support their preferential consideration in complex acetabular reconstructions where durable biologic fixation is essential for long-term success.

## Conclusions

TM acetabular cups demonstrated superior early osseointegration and lower loosening rates compared with conventional revision cups in Paprosky IIC-III defects. These findings suggest that TM implants may offer an advantage in complex acetabular reconstructions requiring durable biological fixation. Future studies with larger cohorts and longer follow-up are needed to confirm these findings and evaluate long-term implant survivorship.
